# Haplotype-sharing analysis for alcohol dependence based on quantitative traits and the Mantel statistic

**DOI:** 10.1186/1471-2156-6-S1-S75

**Published:** 2005-12-30

**Authors:** Andre Kleensang, Daniel Franke, Inke R König, Andreas Ziegler

**Affiliations:** 1 Institute of Medical Biometry and Statistics, University Hospital Schleswig-Holstein, Campus Lübeck, University at Lübeck, Ratzeburger Allee 160, 23538 Lübeck, Germany

## Abstract

Haplotype-based methods have become increasingly popular in the last decade because shared lengths in haplotypes can be used for disease localization. In this contribution, we propose a novel linkage-based haplotype-sharing approach for quantitative traits based on the class of Mantel statistics which is closely related to the weighted pair-wise correlation statistic. Because these statistics are known to be liberal, we propose a permutation test to evaluate significance. We applied the Mantel statistic to the autosomal data from the genome-wide scan of the Collaborative Study on the Genetics of Alcoholism with the Affymetrix Genotype 10 K array that was provided for the Genetic Analysis Workshop 14. Four regions on chromosome 4, 8, 16, and 20 showed *p*-values less than 0.005 with a minimum *p*-value of < 0.0001 on chromosome 16 (tsc0520638 at 72.8 cM). Three of these four regions located on chromosome 4, 16, and 20 have been reported previously in the Genetic Analysis Workshop 11.

## Background

Haplotype-based methods have become increasingly popular in the last decade because shared lengths in haplotypes can be employed to trace disease loci. Thus, they have the potential to incorporate information on chromosome structure and to handle genetic heterogeneity to an extent that exceeds the feasible limit of allelic analyses. The basic haplotype-sharing method idea has been proposed by te Meerman et al. [1] and further developed by te Meerman et al. [2] and te Meerman and van der Meulen [3] at a time when no dense marker maps were yet available. The method is now, however, of even greater interest due to limited informativity of single-nucleotide polymorphisms (SNPs).

The original haplotype-sharing statistic (HSS) is defined as the standard deviation of shared lengths in unrelated case haplotypes. The HSS approach provides a point-wise significance at each marker under study, and has been applied at previous Genetic Analysis Workshops (GAWs) [4] to investigate association.

In the present contribution, we extend this idea and propose a linkage-based haplotype-sharing Mantel (HSM) statistic for the analysis of quantitative traits on family data. Mantel statistics [5] in the context of haplotype sharing were first used by Beckmann [6]. He defined spatial similarity by the shared length between haplotype pairs and temporal similarity as the phenotypic similarity between pairs. Therefore, it is very similar to the weighted pair-wise correlation (WPC) statistic [7,8], which has previously been used for linkage analysis, allele frequency estimation, and estimation of familial correlations.

In this contribution we apply the HSM statistic to the autosomal data from the genome-wide scan of the Collaborative Study on the Genetics of Alcoholism (COGA) with the Affymetrix Genotype 10 K array that was provided for the GAW14.

## Methods

### Data

The provided COGA data included 1,380 study samples within 143 families. The present analysis was based on the Affymetrix Genotype 10 K array clean dataset and contains 10,810 autosomal SNPs.

Following Zinn-Justin and Abel [9], we defined subjects as affected if they met the DSM-III-R criteria of alcohol dependence and the Feighner criteria for alcoholism (phenotype ALDX1). In the next step, the binary phenotype, denoted ALB1, was derived from the ALDX1 phenotypes considering only extremely affected (defined as "affected" in the data description) and unaffected ("purely unaffected" in data description). Other individuals were considered as unknown ALB1.

As phenotypes for our analysis, Pearson residuals were employed from logistic regression predicting the binary phenotypes from sex and age. In detail, sex was coded 0 for males and 1 for females, and age at examination in years was utilized. All individuals from all families were used for the logistic regression. The residuals were denoted as ALB1R. The final fitted logistic regression model was:



### Haplotype-sharing method using Mantel statistics

Originally, Mantel's space-time clustering statistic had the following form



where *X*_*ij *_defines the spatial similarity and *Y*_*ij *_the temporal similarity for the pair *ij*. For haplotype-sharing analyses, Beckmann [6] replaces the spatial similarity with the shared length between haplotype pairs and the temporal similarity with the phenotypic similarity between these pairs.

This is similar to the WPC statistic in which spatial similarity is replaced by genotypic similarity of related pairs measured in terms of alleles shared identical by descent or identical by state [8]. For the application of the HSM statistic to quantitative traits, we propose the mean corrected product of phenotypes as measure of phenotypic similarity which has been suggested previously in the context of Mantel statistics [7,8] and also for linkage analyses:

*Y*_*ij *_= (*Y*_*i *_- μ)·(*Y*_*j *_- μ).

Here, *Y*_*i *_and *Y*_*j *_were given by the phenotypic values ALB1R for individuals *i *and *j*, respectively. In our analyses, Pearson residuals were employed for phenotypes, thus μ = 0. Their use has been discussed [7,8]. Missing phenotypes were assigned a value of 0 after mean correction and therefore these subjects did not contribute to the HSM statistic but to the permutation procedure. This corresponds to a missing completely at random assumption of phenotypes.

The shared length *X*_*ab*_(ℓ) at marker ℓ between haplotypes *a *and *b *was measured as the number of intervals flanked by markers with the same alleles corrected by the mean shared length observed at marker ℓ in the given data [10]:



Because each individual has 2 haplotypes, labelled 1 and 2, and each pair has 4 different haplotype pairs, the final HSM statistic is given by



where *k *denotes the family, and *i*_*a*_*j*_*b*_*k *the haplotype pair *a b *within person pair *ij *within family *k*. Therefore, pairs were constructed within families only, and parent-offspring pairs were discarded from computations.

### Haplotype estimation

In the first step, allele frequencies at single-marker loci were estimated from all individuals. In the second step, we generated a 64-bit build of GENEHUNTER v2.1\_r5 in order to be able to allocate more than 2 GB of CPU memory [details available upon request]. This allows haplotype estimation in 20-bit pedigrees for data from the Affymetrix 10 K array. The number of bits is given by 2*n-f *with *n *denoting the number of nonfounders and *f *the number of founders, respectively. Larger pedigrees were split into 2 or more branches and considered as independent families. In the third step, we estimated inheritance vectors autosome-wise assuming the marker order as provided for GAW14. We stored the most likely pair of haplotypes of an individual within a family estimated by maximum likelihood across the possible set of inheritance vectors. This estimate ignores linkage disequilibrium information from neighboring markers. However, this additional information may be neglected in our sample because haplotypes can be constructed in extended families from segregation patterns.

### Statistical testing

Because the hypothesis of no clustering is equivalent to the situation that the *X*_*i *_occurs randomly with the *Y*_*i*_, we decided to utilize a Monte Carlo permutation approach to estimate the empirical distribution. For this purpose, we generated replicated datasets by randomly permuting phenotypes among family members within all families keeping haplotype sharings unchanged. The empirical *p*-value was defined as the proportion of replicates that led to a statistic with a value greater than the one obtained given the real data. We are fully aware that our permutation approach destroys residual familiar correlation. However, it is not prone to population stratification, because we do not permute phenotypes across families.

Because of computational limitations the analyses were performed in 2 steps. First, we analyzed all SNPs and estimated empirical *p*-values by 1,000 permutations. Second, the number of permutations was increased to 100,000 for regions including more than 3 SNPs with empirical *p*-values < 0.05. Regions including SNPs with *p*-values < 0.005 after the second step of analysis will be reported as most interesting regions.

## Results

### Results from 1,000 permutations (first step of analysis)

Our results from the first analyze yielded 13 regions including more then 3 SNPs with empirical *p*-values < 0.05. These regions are located on chromosomes 3, 4, 5, 8, 9, 12, 14, 16, and 20 and are shown in Table 1.

### Results from 100,000 permutations (second step of analysis)

**Figure 1:**
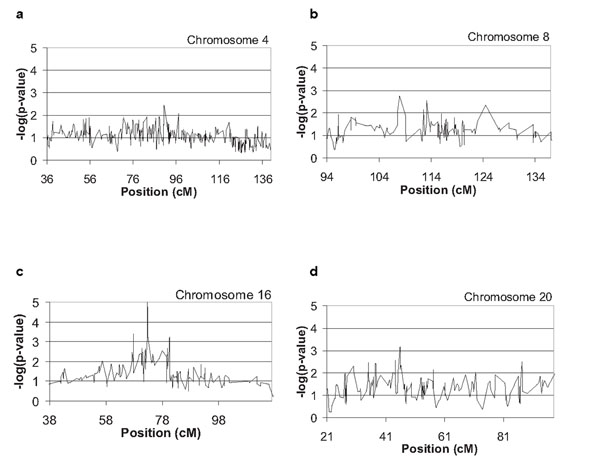
Selected region after second step of analysis for chromosome 4 (a), chromosome 8 (b), chromosome 16 (c), and chromosome 20 (d).

For the 13 regions obtained in the first step of our analysis we increased the number of permutations to 100,000. The results from 4 regions located on chromosome 4, 8, 16, and 20 included SNPs with *p*-values < 0.005. The lowest *p*-value was found at marker tsc0520638 located on chromosome 16 at 72.8 cM. The peak region contains 3 SNPs. All test statistics computed by the permutation approach for the marker tsc0520638 were smaller than that using the real data. If we assume that the true *p*-value corresponds to a LOD = 3, then with 95% confidence the upper limit of the *p*-value is approximately 6.2·10^-5 ^in 100,000 simulations. The results for the 4 chromosomes are shown in Figure 1.

## Discussion

Our analysis uses a new Mantel based haplotype-sharing approach for quantitative traits within family data applied to the autosomal data from the genome-wide scan of the COGA with the Affymetrix Genotype 10 K array that was provided for GAW14. Our method is similar Beckmann's haplotype-sharing approach [6]; however, we employ family data. Furthermore, we extended the method to the analysis of quantitative traits. We permuted phenotypes within families. Our approach therefore may be considered as a linkage method and is not prone to population stratification, because we do not permute phenotypes across families. Whether the permutation procedure destroys heritability resulting in a compound hypothesis remains to be clarified in further analyses.

In the analyses, we identified 3 linkage regions that had been reported previously in GAW11. More precisely, Daw et al. [11] and Jacobs et al. [12] had detected the region on chromosome 4, Kovac et al. [13] and Macciardi et al. [14] on chromosome 16, and Palmer et al. [15] and Zinn-Justin et al. [9] on chromosome 20. We detected an additonal signal with our method on chromosome 8, which has not been reported before. The region contains approximately 35 genes with regulatory functions. This finding needs further investigation, preferably in an independent validation study.

## Abbreviations

COGA: Collaborative Study on the Genetics of Alcoholism

GAW: Genetic Analysis Workshop

HSM statistic: Haplotype-sharing Mantel statistic

HSS: Haplotype-sharing statistic

SNP: Single-nucleotide polymorphisms

WPC: Weighted pair-wise correlation

## Authors' contributions

AZ had the original idea for the study and provided intellectual input. DF did the programming. AK and IRK performed the analyses and wrote the first draft of the manuscript. All authors read and approved the final manuscript.

## Figures and Tables

**Table 1:** Selected regions after first step of analysis (1,000 permutations)

Chromosome	Marker	Position (cM)	Marker	Position (cM)
3	tsc0041431	121.20	tsc0779613	124.16
4	tsc0042111	0.45	tsc0584121	22.22
4	tsc0275833	35.93	tsc0047661	139.76
5	tsc1534591	78.58	tsc0313571	92.44
8	tsc0534320	0.42	tsc0538934	9.78
8	tsc0668824	18.76	tsc0046166	32.15
8	tsc0590540	93.37	tsc0945708	137.81
9	tsc0041933	98.70	tsc0596780	128.25
12	tsc0966917	49.55	tsc0690704	55.58
14	tsc1043437	4.21	tsc0549368	15.01
16	tsc0050233	37.68	tsc0564806	117.98
20	tsc0603237	20.97	tsc0594829	98.63
22	tsc1293972	2.69	tsc0273461	12.58

Regions selected from first scan step which includes more then 3 SNPs with *p*-values < 0.05.
